# Triterpenoids from the Roots of *Rhaphiolepis indica* var. *tashiroi* and Their Anti-Inflammatory Activity

**DOI:** 10.3390/ijms14058890

**Published:** 2013-04-24

**Authors:** Chu-Hung Lin, Hsun-Shuo Chang, Hsiang-Ruei Liao, Ih-Sheng Chen, Ian-Lih Tsai

**Affiliations:** 1 School of Pharmacy, College of Pharmacy, Kaohsiung Medical University, Kaohsiung 80708, Taiwan; E-Mail: u96830006@kmu.edu.tw (C.-H.L.); 2 Graduate Institute of Natural Products, College of Pharmacy, Kaohsiung Medical University, Kaohsiung 80708, Taiwan; E-Mail: hschang@kmu.edu.tw (H.-S.C.); 3 Graduate Institute of Natural Products, Chang Gung University, Taoyuan 33302, Taiwan; E-Mail: liaoch@mail.cgu.edu.tw

**Keywords:** *Rhaphiolepis indica* var*. tashiroi*, Rosaceae, roots, triterpenoids, anti-inflammatory activity, inhibition of superoxide production

## Abstract

Two new triterpenoids, 2α,3β-dihydroxyolean-11,13(18)-dien-19β,28-olide (**1**) and 3β,5β-dihydroxyglutinol (**2**), together with eight known compounds (**3**–**10**) were isolated from the roots of *Rhaphiolepis indica* var*. tashiroi* (Rosaceae). The structures of **1**–**10** were determined by spectroscopic techniques. Among these isolates, 2α,3β-dihydroxyolean-13(18)-en-28-oic acid (**9**) exhibited inhibitory effect on *N*-formyl-methionyl-leucyl-phenylalanine (fMLP)-induced superoxide production, with an IC_50_ value of 16.50 μM.

## 1. Introduction

**Figure 1:**
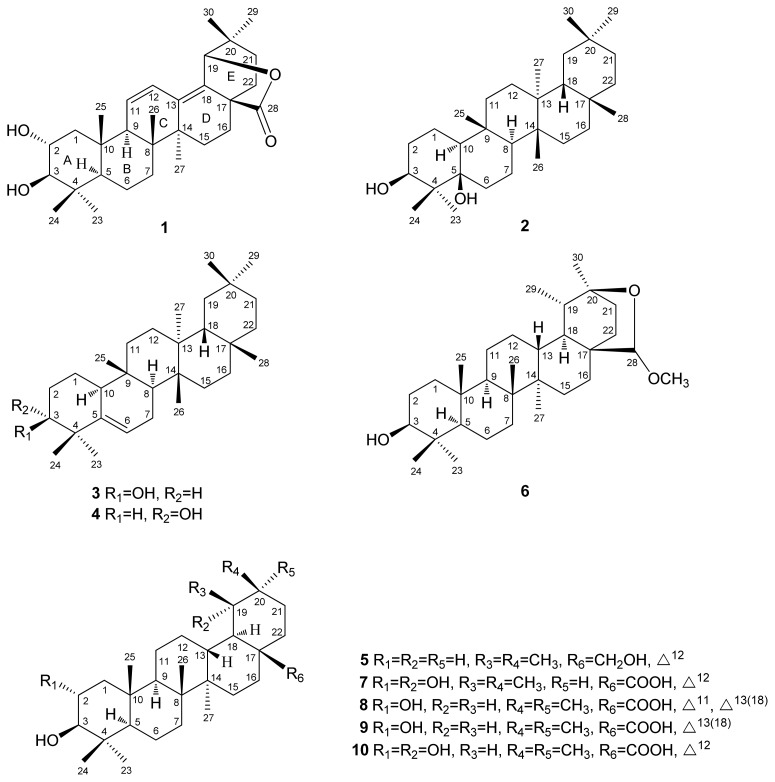
Structures of compounds **1**–**10**.

Under a screening program of Formosan plants on inhibition of *N*-formyl-methionyl-leucyl-phenylalanine (fMLP) induced superoxide production assay, a methanolic extract of the roots of *Rhaphiolepis indica* var. *tashiroi* (Rosaceae) has shown suppression of *N*-formyl-methionyl-leucyl-phenylalanine (fMLP)-induced superoxide production with no cytotoxicity against neutrophils. *R. indica* var. *tashiroi* is an evergreen shrub or small tree, which is distributed in countries throughout Asia, including India, southern China, the Ryukyus, Korea, and Taiwan [1]. We previously reported six new compounds, including four dibenzofurans, 2-hydroxy-3,4,6-trimethoxydibenzofuran, 2-hydroxy-3,4,9-trimethoxydibenzofuran, 2-hydroxy-3,4,6,9-tetramethoxydibenzofuran, and 1,2-methylenedioxy-3,4,6-trimethoxydibenzofuran, two new biphenyls, 3-hydroxy-2′,5-dimethoxybiphenyl and 2′,3-dihydroxy-5-methoxybiphenyl, and one known 3-hydroxy-5-methoxybiphenyl from the roots of *R. indica* var*. tashiroi* [2]. Continuous study from the roots of this plant led to the isolation of two new triterpenoids, one oleanane-type 2α,3β-dihydroxy-olean-11,13(18)-dien-19β,28-olide (**1**), and one glutinane-type 3β,5β-dihydroxyglutinol (**2**), together with eight known compounds (**3**–**10**) (Figure 1). This paper describes the structural elucidations and inhibition of superoxide production activity of these isolates.

## 2. Results and Discussion

### 2.1. Structure Elucidation

**Figure 2:**
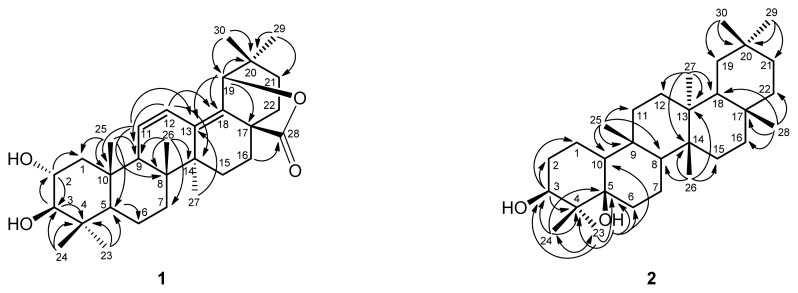
Key HMBC (↷) connectivities for compounds **1** and **2**.

**Figure 3:**
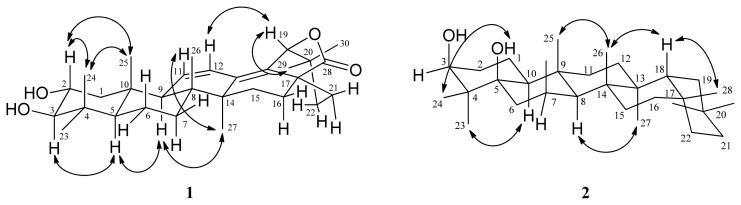
Key NOESY (↷) connectivities for compounds **1** and **2**.

Compound **1** was obtained as amorphous powder. ESIMS (*m/z* 491 [M + Na]^+^) and HRESIMS (*m/z* 491.3136 [M + Na]^+^) analysis established the molecular formula of **1** as C_30_H_44_O_4_. The IR absorption bands suggested the presence of hydroxy (3440 cm^−1^), γ-lactonic carbonyl (1767 cm^−1^), and olefinic (1630, 1600 cm^−1^) groups. The ^1^H NMR spectrum (Table 1) of **1** indicated seven methyl singlets (δ_H_ 0.76, 0.82, 0.95, 1.01, 1.02, 1.04, and 1.10), and a pair of *cis* olefinic protons at δ 5.77 (1H, dd, *J =* 10.2, 2.0 Hz, H-11) and 6.15 (1H, dd, *J =* 10.2, 2.8 Hz, H-12). The ^13^C NMR and DEPT spectra revealed that **1** had 30 carbons. Two tertiary olefinic carbons at δ 123.3 (C-12) and 129.1 (C-11), and two quaternary olefinic carbons at δ 132.9 (C-18) and 134.7 (C-13) indicated the presence of two double bonds. Two oxymethine carbons at δ 68.8 and 83.9, and a γ-lactonic carbonyl carbon at δ 178.1 supported the proposed structure as **1**, which was similar to that of camaldulenic acid (**8**) with oleanane-type skeleton [3]. The major difference was a γ-lactonic carbonyl group in ring E in **1** instead of the COOH group in **8**. The observed oxygen-bearing tertiary carbon signal at C-19 (δ_C_ 85.0) as well as the HMBC correlations from H-19 (δ 4.72, s) to C-13, C-17, C-18, C-20, and C-21 indicated a five-membered lactone ring between C-17 and C-19 which are also supported by IR absorption and nine degrees of unsaturation. The locations of two double bonds at Δ^11^ and Δ^13(18)^ were determined by the HMBC correlations from H-11 to C-8, C-10, and C-13, from H-12 to C-9, C-14, and C-18, from H-27 to C-13, and from H-19 to C-13 and C-18 (Figure 2). The relative configurations of **1** were determined through inspection of the NOESY spectrum (Figure 3). The NOESY cross-peaks of H-2/H-25 and H-3/H-5 established the β-orientation of H-2 and α-orientation of H-3 in *trans* A/B ring junction [4]. Thus, **1** was elucidated as 2α,3β-dihydroxyolean-11,13(18)-dien-19β,28-olide.

Compound **2** was isolated as amorphous powder. The HRESIMS analysis of **2** revealed an [M + Na]^+^ ion peak at *m/z* 467.3862 (calcd. 467.3865), which corresponds to the molecular formula C_30_H_52_O_2_, accounting for five degrees of unsaturation. The IR absorption bands showed a hydroxy group at 3400 cm^−1^. The ^1^H NMR spectrum (Table 1) revealed eight methyl groups (δ_H_ 0.89, 0.94, 0.95, 0.97, 0.99, 1.01, 1.01, and 1.17). The NMR data of **2** resembles to those of glutinol (**3**) with glutinane-type skeleton. The major differences between **2** and **3** were having a quaternary 5-OH group (δ_H_ 1.10; δ_C_ 77.4) and a C-6 methylene (δ_H_ 1.55, 1.77; δ_C_ 34.4) in **2** instead of trisubstituted olefinic signals at C-5 and C-6 in **3**. The ^13^C NMR signal atδ_C_ 73.5 and its corresponding proton signal at δ_H_ 3.76 (1H, dd, *J* = 11.2, 4.0 Hz, H-3), were assigned as C-3 and H-3α, respectively [5]. Moreover, the HMBC correlations (Figure 2) from H-3 to C-1, C-2, and C-4, from H-23 to C-3, C-4 C-5 and C-24, from H-24 to C-3, C-4, C-5, and C-23, and from OH-5 to C-4, C-5, C-6, and C-10, suggested that two hydroxy groups were attached to C-3 and C-5, respectively. The relative configurations of **2** were determined through inspection of the NOESY spectrum (Figure 3). The several key NOESY correlations (H-24/OH-5; H-23/H-10; H-8/H-27; H-25/H-26; H-18/H-26; H-18/H-28) suggested that the β-axial orientation of OH-3 and α-orientation of H-10 in *trans* A/B ring junction (Figure 3). On the basis of these data, **2** was established as 3β,5β-dihydroxyglutinol.

The known compounds, glutinol (**3**) [6], 5(6)-gluten-3α-ol (**4**) [7], uvaol (**5**) [8], 20β,28-epoxy-28α-methoxytaraxasteran-3β-ol (**6**) [9], tormentic acid (**7**) [10], camaldulenic acid (**8**) [3], 2α,3β-dihydroxyolean-13(18)-en-28-oic acid (**9**) [4], arjunic acid (**10**) [8] were identified by comparison of their physical and spectroscopic data with values reported in the literatures.

### 2.2. Inhibition of Superoxide Production Activities

The inhibition of superoxide production of the compounds *in vitro* was estimated by an assay on inhibitory activity of induction of the *N*-formyl-methionyl-leucyl-phenylalanine (fMLP)-induced generation of the superoxide anion, an inflammatory mediator produced by neutrophils. The clinical anti-inflammatory agent ibuprofen was used as the positive control. The results are shown in Table 2. The effects of compound **9** (IC_50_ 16.50 ± 0.56 μM) on fMLP-induced superoxide generation was more potent than that of ibuprofen (IC_50_ 27.53 ± 3.58 μM). However, compounds **1**, **2**, and **8** showed weaker activities than that of ibuprofen with IC_50_ values of 98.37, 56.08 and 89.42 μM.

## 3. Experimental Section

### 3.1. General Experimental Procedures

All melting points were measured on a Yanaco micro-melting apparatus and were uncorrected. Optical rotations were measured on a Jasco P-1020 digital polarimeter. The UV spectra were obtained with a Jasco V-530 UV/VIS spectrophotometer, and IR spectra (KBr or neat) were taken on a Perkin-Elmer System 2000 FT-IR spectrometer. 1D (^1^H, ^13^C, DEPT) and 2D (COSY, NOESY, HSQC, HMBC) NMR spectra using CDCl_3_ or CD_3_OD as solvent were recorded on Varian Germini 2000-200 (200 MHz for ^1^H NMR, 50 MHz for ^13^C NMR), Varian Unity Plus 400 (400 MHz for ^1^H NMR, 100 MHz for ^13^C NMR) and Varian VNMRS-600 (600 MHz for ^1^H NMR, 150 MHz for ^13^C NMR) spectrometers. Chemical shifts were internally referenced to the solvent signals in CDCl_3_ (^1^H, δ 7.26; ^13^C, δ 77.0) or CD_3_OD (^1^H, δ 3.31; ^13^C, δ 49.0), with TMS as the internal standard. ESIMS were obtained on an API 3000 mass spectrometer (Applied Biosystems) and HRESIMS on a Bruker Daltonics APEX II 30e mass spectrometer. Silica gels (70–230, 230–400 mesh) (Merck) were used for column chromatography (CC), and silica gel 60 F-254 (Merck) was used for analytical and preparative TLC. A spherical C18 100 Å column (20–40 μM) (Silicycle) was used for medium-pressure liquid chromatography.

### 3.2. Plant Material

The dried roots of *R. indica* var. *tashiroi* were collected at Wutai, Pingtung County, Taiwan, in September 2007, and identified by one of the authors (I.-S.C.). A voucher specimen (Chen 6060) was deposited in the Herbarium of the School of Pharmacy, College of Pharmacy, Kaohsiung Medical University, Kaohsiung, Taiwan, China.

### 3.3. Extraction and Isolation

Dried roots (32.8 kg) of *R. indica* var. *tashiroi* were extracted three times with cold MeOH (40 L) to yield a MeOH extract (1.9 kg), which was partitioned using EtOAc–H_2_O (1:1; 2 L × 3) to produce an EtOAc-soluble fraction (600 g) and an H_2_O-soluble fraction. The H_2_O-soluble fraction was partitioned in *n*-BuOH:H_2_O (1:1; 3L × 3) to obtain an *n*-BuOH-soluble fraction (700 g) and an H_2_O-soluble fraction (400 g). The EtOAc-soluble fraction (100 g) was subjected to silica gel column chromatography (CC) using *n*-hexane as the primary eluting solvent and gradually increasing the eluent polarity with EtOAc and MeOH to produce 12 fractions (A-1–A-12). Fractions A-5 and A-7 showed anti-inflammatory activity. Fraction A-4 (590 mg) was subjected to MPLC using a mixture of *n*-hexane:EtOAc (15:1) to obtain seven fractions (A-4-1–A-4-7). Fraction A-4-3 (126 mg) was separated by MPLC using a mixture of *n*-hexane:CH_2_Cl_2_ (1:1) to obtain four fractions (A-4-3-1–A-4-3-4). Fraction A-4-3-1 (25.9 mg) was separated by MPLC using a mixture of *n*-hexane:CH_2_Cl_2_ (2:1) to afford **3** (21.9 mg). Fraction A-4-4 (94.5 mg) was separated by MPLC using a mixture of *n*-hexane:CH_2_Cl_2_ (1:1) to obtain nine fractions (A-4-1–A-4-9). Fraction A-4-4-7 (19.7 mg) was separated by MPLC using a mixture of *n*-hexane:CH_2_Cl_2_ (1:2) to afford **4** (9.5 mg). Fraction A-6 (1.46 g) was subjected to silica gel CC using a mixture of *n*-hexane:acetone (7:1) as the eluent to yield eight fractions (A-6-1–A-6-8). Fraction A-6-4 (388 mg) was applied to MPLC using a mixture of *n*-hexane:acetone (10:1) to yield four fractions (A-6-4-1–A-6-4-4). Fraction A-6-4-1 (199 mg) was separated by MPLC (RP-18) using a mixture of acetone:H_2_O (3:1) to produce **5** (10.3 mg). Fraction A-7 (685 mg) was subjected to MPLC using a mixture of *n*-hexane:acetone (8:1) to obtain seven fractions (A-7-1–A-7-7). Fraction A-7-6 (77.4 mg) was separated by MPLC using a mixture of CH_2_Cl_2_:EtOAc (20:1) to obtain **2** (5 mg), Fraction A-7-7 (54.8 mg) was subjected to MPLC using a mixture of *n*-hexane:acetone (3:1) to obtain seven fractions (A-7-7-1–A-7-7-7). Fraction A-7-7-3 (13.9 mg) was purified by preparative TLC (RP-18) developed with acetone:H_2_O (1:6) to yield **6** (2.9 mg). Fraction A-9 (697 mg) was applied to MPLC using a mixture of CH_2_Cl_2_:MeOH (50:1) to provide six fractions (A-9-1–A-9-6). Fraction A-9-2 (98.2 mg) was subjected to MPLC (RP-18) using a mixture of acetone:H_2_O (2:1) to obtain **1** (4.6 mg). Fraction A-9-4 (139.9 mg) was applied to MPLC (RP-18) using a mixture of MeOH:H_2_O (1:1) to provide **7** (68.2 mg), **8** (9.3 mg), and **9** (74.7 mg). Fraction A-10 (811 mg) was separated by MPLC (RP-18) using a mixture of MeOH:H_2_O (3:1) to produce 12 fractions (A-10-1–A-10-12). Fraction A-10-12 (23.1 mg) was subjected to MPLC using a mixture of CH_2_Cl_2_:MeOH (20:1) to obtain **10** (7.6 mg).

2α,3β-Dihydroxyolean-11,13(18)-dien-19β,28-olide (**1**): amorphous powder; ${\left[\alpha \right]}_{\text{D}}^{24}$ +49.5 (*c* 0.09, CHCl_3_); IR (KBr) ν_max_ cm^−1^: 3440 (OH), 1767 (γ-lactonic C=O), 1630, 1600; for ^1^H and ^13^C NMR spectroscopic data, see Table 1; ESIMS: *m/z* 491 [M + Na]^+^; HRESIMS: *m/z* 491.3136 [M + Na]^+^ (calcd. for C_30_H_44_O_4_Na, 491.3137).

3β,5β-Dihydroxyglutinol (**2**) : amorphous powder; ${\left[\alpha \right]}_{\text{D}}^{24}$ +7.6 (*c* 0.11, CHCl_3_); IR (KBr) ν_max_ cm^−1^: 3400 (OH); for ^1^H and ^13^C NMR spectroscopic data, see Table 1; ESIMS: *m/z* 467 [M + Na]^+^; HRESIMS: *m/z* 467.3862 [M + Na]^+^ (calcd. for C_30_H_52_O_2_Na, 467.3865).

### 3.4. Inhibition of Superoxide Production Assay

#### 3.4.1. Evaluation of O_2_^•−^ Release by Human Neutrophils

The anti-inflammatory effects of the compounds isolated from the roots of *R. indica var. tashiroi* were evaluated by measuring the inhibition of superoxide anion production. Superoxide anion production was tested using continuous spectrophotometric analysis of ferricytochrome *c* reduction by an isolated preparation of human neutrophils.

#### 3.4.2. Preparation of Human Neutrophils

Human neutrophils from the venous blood of healthy [11], adult volunteers (20–28 years old) were isolated using a standard method of dextran sedimentation, before centrifugation in a Ficoll Hypaque gradient and hypotonic lysis of the erythrocytes [12]. The purified neutrophils containing >98% viable cells, as determined by the Trypan blue exclusion method, were resuspended in a Ca^2+^ (1 mM) Hank’s balanced salt solution (pH 7.4) and maintained at 4 °C until use.

#### 3.4.3. Measurement of O_2_^•−^ Generation

The assay for measuring O_2_^•−^ generation was based on the superoxide dismutase (SOD)-inhibitable reduction of ferricytochrome *c* [13]. Briefly, neutrophils (1 × 10^6^ cells/mL), pretreated with various concentrations of the test compounds for 5 min at 37 °C, were stimulated with fMLP (1 μmol/L) in the presence of ferricytochrome *c* (0.5 mg/mL). Extracellular O_2_^•−^ production was assessed using a UV spectrophotometer at 550 nm (Hitachi, UV-3010). The percentage of superoxide inhibition by the test compound was calculated as {[(control − resting) − (compound − resting)]/(control − resting)} × 100. SigmaPlot software was used to determine the IC_50_ value.

#### 3.4.4. Statistical Analysis

The results are expressed as the means ± SEM, and comparisons were made with the Student’s *t* test. A probability of 0.05 or less was considered significant.

## 4. Conclusions

There are about five Rhaphiolepis species in India, southeastern and eastern Asia and one species with three varieties in Taiwan [1]. The leaves of *R. umbellata* were previously reported to have dibenzofurans [14], biphenyls [14–16] as phytoalexin with antifungal activity, and its bark contained flavanol glycosides [17] and procyanidins [18]. Two new triterpenoids were isolated from the roots of *R. indica* var. *tashiroi*. To our knowledge, this is the first report of triterpenoids from the Rhaphiolepis plants. The inhibition of superoxide production of the Rhaphiolepis genus has never been examined by other research groups. However, we previously reported anti-inflammatory biphenyls and dibenzofurans from the roots of this Formosan plant [2]. Compound **9** was previously reported to show the anti-inflammatory activity [4]. In this study, **9** showed stronger inhibition of superoxide production (IC_50_ 16.50 ± 0.56 μM) than ibuprofen (IC_50_ 27.53 ± 3.58 μM) suggesting the possiblity of developing a new anti-inflammatory agent. This result implied that the anti-inflammatory mechanism of action is worth studying further.

## Figures and Tables

**Table 1:** ^1^H (400 MHz) and ^13^C (100 MHz) NMR data of **1** and **2** (CDCl_3_).

position	1		2	
	
	δ_C_	δ_H_ (*J* in Hz)	δ_C_	δ_H_ (*J* in Hz)
1	41.6 (CH_2_)	0.97, m; 2.24, dd (12.4, 4.4)	19.1 (CH_2_)	1.52, m; 1.56, m
2	68.8 (CH)	3.77, ddd (9.6, 4.4, 2.0)	30.6 (CH_2_)	1.39, m; 1.78, m
3	83.9 (CH)	3.03, d ( 9.6)	73.5 (CH)	3.76, dd (11.2, 4.0)
4	39.2 (C)	–	43.9 (C)	–
5	54.9 (CH)	0.91, m	77.4 (C)	–
6	18.1 (CH_2_)	1.44, m	34.4 (CH_2_)	1.55, m; 1.77, m
7	33.0 (CH_2_)	1.39, m; 1.46, m	17.2 (CH_2_)	1.45, m; 1.50, m
8	41.0 (C)	–	52.5 (CH)	1.24, m
9	52.8 (CH)	2.14, br s	36.8 (C)	–
10	37.9 (C)	2.40, m	50.9 (CH)	1.31, m
11	129.1 (CH)	5.77, dd (10.2, 2.0)	34.9 (CH_2_)	1.18, m; 1.54, m
12	123.3 (CH)	6.15, dd (10.2, 2.8 )	30.3 (CH_2_)	1.31, m; 1.39, m
13	134.7 (C)	–	38.2 (C)	–
14	40.6 (C)	–	39.5 (C)	–
15	25.5 (CH_2_)	1.27, m; 1.39, m	32.5 (CH_2_)	1.28, m; 1.51, m
16	24.2 (CH_2_)	2.33, ddd (14.4, 5.2, 2.6)	39.2 (CH_2_)	0.92, m; 1.48, m
17	44.0 (C)	–	29.8 (C)	–
18	132.9 (C)	–	42.7 (CH)	1.54. m
19	85.0 (CH)	4.72, s	32.8 (CH_2_)	1.28, m; 1.51, m
20	35.7 (C)	–	28.2 (C)	–
21	32.6 (CH_2_)	1.45, m; 1.62, m	35.3 (CH_2_)	1.18, m; 1.38, m
22	34.5 (CH_2_)	1.63, m; 1.83, m	35.9 (CH_2_)	1.37, m; 1.56, m
23	28.3 (CH_3_)	1.04, s	19.2 (CH_3_)	0.97, s
24	16.3 (CH_3_)	0.82, s	16.5 (CH_3_)	0.89, s
25	19.1 (CH_3_)	1.02, s	17.1 (CH_3_)	0.95, s
26	16.9 (CH_3_)	0.76, s	20.4 (CH_3_)	1.01, s
27	19.4 (CH_3_)	1.01, s	18.7 (CH_3_)	1.01, s
28	178.1 (C)	–	32.1 (CH_3_)	1.17, s
29	27.8 (CH_3_)	1.10, s	35.0 (CH_3_)	0.94, s
30	23.3 (CH_3_)	0.95, s	31.8 (CH_3_)	0.99, s
OH-5	–	–	–	1.10, br s check

**Table 2:** IC_50_ Values for the isolates of the roots of *R. indica* var*. tashiroi* in the inhibition on *N*-formyl-methionyl-leucyl-phenylalanine (fMLP)-induced superoxide generation in human neutrophils.

Compounds	IC_50_ (μM)^a^
2α,3β-dihydroxyolean-11,13(18)-dien-19β,28-olide (**1**)	98.37 ± 6.84
3β,5β-dihydroxyglutinol (**2**)	56.08 ± 0.57
glutinol (**3**)	>100
5(6)-gluten-3α-ol (**4**)	>100
uvaol (**5**)	>100
20β,28-epoxy-28α-methoxytaraxasteran-3β-ol (**6**)	>100
tormentic acid (**7**)	>100
camaldulenic acid (**8**)	89.42 ± 4.26
2α,3β-dihydroxyolean-13(18)-en-28-oic acid (**9**)	16.50 ± 0.56
Ibuprofen^b^	27.53 ± 3.58

a The IC_50_ values were calculated from the slopes of the dose–response curves. The values are expressed as the means ± standard errors of the means (SEM) of three independent experiments.

b Positive control.
